# CHIP, a carboxy terminus HSP-70 interacting protein, prevents cell death induced by endoplasmic reticulum stress in the central nervous system

**DOI:** 10.3389/fncel.2014.00438

**Published:** 2015-01-09

**Authors:** Felipe Cabral Miranda, Juliana Adão-Novaes, William W. Hauswirth, Rafael Linden, Hilda Petrs-Silva, Luciana B. Chiarini

**Affiliations:** ^1^Laboratório de Neurogênese, Departamento de Neurobiologia, Instituto de Biofísica Carlos Chagas Filho, Universidade Federal Do Rio de JaneiroRio de Janeiro, Brazil; ^2^Retinal Gene Therapy Group, Department of Ophthalmology, University of FloridaGainesville, FL, USA

**Keywords:** UPR signaling pathways, neurodegeneration, AAV-vectors, Hippocampus, ER stress, CHIP

## Abstract

Endoplasmic reticulum (ER) stress and protein misfolding are associated with various neurodegenerative diseases. ER stress activates unfolded protein response (UPR), an adaptative response. However, severe ER stress can induce cell death. Here we show that the E3 ubiquitin ligase and co-chaperone Carboxyl Terminus HSP70/90 Interacting Protein (CHIP) prevents neuron death in the hippocampus induced by severe ER stress. Organotypic hippocampal slice cultures (OHSCs) were exposed to Tunicamycin, a pharmacological ER stress inducer, to trigger cell death. Overexpression of CHIP was achieved with a recombinant adeno-associated viral vector (rAAV) and significantly diminished ER stress-induced cell death, as shown by analysis of propidium iodide (PI) uptake, condensed chromatin, TUNEL and cleaved caspase 3 in the CA1 region of OHSCs. In addition, overexpression of CHIP prevented upregulation of both CHOP and p53 both pro-apoptotic pathways induced by ER stress. We also detected an attenuation of eIF2a phosphorylation promoted by ER stress. However, CHIP did not prevent upregulation of BiP/GRP78 induced by UPR. These data indicate that overexpression of CHIP attenuates ER-stress death response while maintain ER stress adaptative response in the central nervous system. These results indicate a neuroprotective role for CHIP upon UPR signaling. CHIP emerge as a candidate for clinical intervention in neurodegenerative diseases associated with ER stress.

## Introduction

Cells present ingenious mechanisms of protein quality control to avoid aggregation of unfolded proteins and to guarantee correct conformation of newly synthesized molecules (Tyagi, 2012). These processes take place in the cytosol, mitochondria and endoplasmic reticulum (ER). In the endoplasmic reticulum, ER chaperones bind to nascent polypeptide chains and assist protein folding, while misfolded proteins are directed to proteasomal degradation in the cytosol by endoplasmic reticulum-associated degradation (ERAD) (Hebert and Molinari, 2007; Tsai and Weissman, 2011).

Stressful environmental conditions such as increased temperatures, hypoxia, oxidative stress and altered glucose metabolism affect protein folding and derail the homeostasis of the endoplasmic reticulum. This condition, known as ER stress (Sherman and Goldberg, 2001; Hetz, 2012; Yu et al., 2012), activates three distinct signaling pathways: PERK, IRE1, and ATF6, collectively known as the unfolded protein response (UPR) (Jäger et al., 2012). UPR activates mechanisms related to ER buffering, such as upregulation of genes involved in protein folding, degradation, and redox regulation, and also transient inhibition of global protein translation, which tend to oppose the higher demand for ER function (Hetz, 2012).

Although the UPR grants both adaptive and protective responses, unfolded and misfolded proteins may lead to ER stress-induced cell death, which confers a dual role to both ER stress and UPR (Morimoto and Yves, 2013; Lu et al., 2014). Interestingly, various neurodegenerative conditions, including Alzheimer's, Parkinson's, and Huntington's diseases, amyotrophic lateral sclerosis, as well as acute events such as ischemia or brain trauma are associated with UPR (Halliday and Mallucci, 2014). Upregulated CHOP and p53 were described as mediators of an ER stress-induced cell death response (Li et al., 2006). Besides, the phosphorylation of eIF2-α by PERK was recently described as a critical step in an animal model of prion disease (Moreno et al., 2012; Moreno and Tiffany-Castiglioni, 2014). Thus, the finding of elements that control or prevent cell death mediated by ER stress may offer new avenues for the management of various diseases in the central nervous system.

Previous publications have addressed neuroprotective function of the protein CHIP (carboxyl terminus of the Hsc70-interacting protein) in heat shock stress (Dai et al., 2003), tauopathies (Petrucelli et al., 2004; Dickey et al., 2007a), and oxidative stress (Lee et al., 2013). Furthermore, deficiency of CHIP decreases longevity, accelerates aging together with altered protein quality control and increased oxidative stress (Min et al., 2008). CHIP has recently emerged as a central player in the selection and degradation of unfolded proteins that interact with the chaperoning complex HSP-70/90 (Dickey et al., 2007b). CHIP acts as a co-chaperone through its tetratricopeptide (TPR) domain, and directly modulates the interaction of HSP-70 with its client proteins. In addition, CHIP has an U-BOX domain in its carboxyl-terminal portion, which confers E3 ubiquitin ligase activity, thus helping target client proteins, inclusive of HSP-70, for proteasomal degradation (Connell et al., 2001; Petrucelli et al., 2004). Thus, CHIP provides a direct link between the HSP chaperone and Ubiquitin Proteasome systems, and may also contribute to the balance of protein folding and degradation (McClellan and Frydman, 2001). Although ubiquitin/proteasome-dependent protein degradation occurs in the cytosol and nucleus, there is a connection between the protein quality control in the ER and in the cytoplasm (Plemper and Wolf, 1999; Menéndez-Benito et al., 2005). Still, there is no description of the effect of CHIP upon protein quality control in the ER.

Despite the described function of CHIP in the folding and degradation of proteins, its relation with the UPR and ER-stress-induced cell death has not been investigated in nervous tissue. This study aimed to evaluate the role of CHIP upon the regulation of ER stress-induced cell death in cultures of hippocampal slices from juvenile rats, a model widely used in studies of the mechanisms of neurodegeneration (Kosuge et al., 2008).

## Materials and methods

### Organotypic hippocampal slice cultures

All procedures were approved by the University Ethics Committee under protocol IBCCF 172. Hippocampal slices were produced as described previously, with slight modifications (Stoppini et al., 1991). Briefly, male Lister Hooded rats at postnatal day 6–7 were decapitated and hippocampi were dissected under sterile conditions in ice cold Hank's Balanced Salt Solution (HBSS, Gibco). Hippocampi were sliced transversally at 400 μm with a McIlwain Tissue Chopper (Mickle Laboratories). Slices were then transferred to 30-mm-diameter membrane inserts (Millicell, Millipore) and cultured for 14 days in 6-well culture trays with 1 mL of medium per well. Culture medium included 50% minimum essential medium (MEM), 25% HBSS, 25% heat inactivated horse serum, 1% penicillin, 1% streptomycin in addition to 25 mM HEPES, 36 mM D-glucose, 4 mM Na_2_HCO_3_, pH 7.3. Cultures were kept in a humidified incubator at 37°C and 5% CO_2_. Medium was changed every 3 days.

### Production of recombinant adeno-associated viral vector serotype-8 (rAAV8)

Vector preparations were produced by the plasmid co-transfection method as shown previously (Petrs-Silva et al., 2011). Briefly, the crude iodixanol fraction with rAAV vectors was further purified and concentrated by column chromatography on a 5-ml HiTrap Q Sepharose column using an AKTA FPLC system (Amersham Biosciences, Piscataway, NJ). The vector was eluted from the column using 215 mM NaCl, pH 8.0, and the vector containing fractions were collected, pooled, concentrated, and buffer exchanged into Alcon BSS with 0.014% Tween 20, using a Biomax 100 K concentrator (Millipore, Billerica, MA). The titer of DNase-resistant vector genomes was measured by real-time PCR relative to a standard. Finally, the purity of the vector was validated by silver-stained sodium dodecyl sulfate–polyacrylamide gel electrophoresis, assayed for sterility and lack of endotoxin, and then aliquoted and stored at −80°C. Each vector contained the genome encoding green fluorescent protein (GFP) or human CHIP under the control of a ubiquitous chicken beta-actin (CBA) promoter.

### Drug and viral vector treatments

Recombinant adeno-associated viral vector serotype 8 (rAAV8) was used to promote CHIP overexpression in hippocampal tissue and rAAV8GFP was used as a control. Hippocampal slices were infected with 10E9 vector genomes. A total of 1 μl virus solution at 1.36_12_ VG/mL was applied directly to the top of each hippocampal slice, at 30 min of culture (day 0). All analysis were done 14 days after infection. Tunicamycin (TN, Sigma) an inhibitor of N-glycosylation widely used as an ER stress inducer, was diluted in DMSO and added to culture medium. Slices were incubated with the inhibitor at day 13, in various concentrations for 24 h, completing 14 days *in vitro*. Control slices were incubated with DMSO (0.016%) only.

### Assessment of necrotic cell death

Propidium iodide (PI) at concentration of 5 μg/mL was added to the organotypic hippocampal slices cultures (OHSCs) after the experimental procedures. Cell death by necrosis was identified by the uptake of PI (Macklis and Madison, 1990; Raval et al., 2003; Kosuge et al., 2008). Images were obtained with an inverted epi-illumination fluorescence microscope (Zeiss, MRMm Rev3) with a cold-CCD camera system (Axiocam) at 4x magnification. The same exposure time was used for all independent experiments. PI fluorescence was quantitatively analyzed using ImageJ Software and was expressed as a percentage of the maximum fluorescence (Ff), obtained after tissue fixation with 4% paraformaldehyde (4% PF).

**Cell death (%) = (F − F0)/(Ff − F0) × 100**: where **F** is the PI fluorescence of slices measured at 24 h of drug exposure; **F0** is the background fluorescence prior to treatment.

### TUNEL assay and nuclear staining

The ApopTag® *In Situ* Apoptosis Detection Kit was used for TUNEL assay. Slices not treated with PI were fixed with paraformaldehyde (PF) 4% for 2 h, and then washed with PBS. The slices were then incubated in 1% Triton X-100 in PBS for 45 min. After 3 PBS washes of 5 min, they were incubated with Equilibration Buffer for 10 min at room temperature, and then with 30% of TdT Enzyme and 70% of Reaction Buffer for 2 h at 37°C. Slices were then incubated with Stop/Wash Buffer for 10 min at room temperature and washed with PBS. After incubation with Anti-Digoxigenin-Fluorescein (47%), Blocking Solution (53%) plus TO-PRO3 (1:1000) for 1 h, slices were again washed with PBS and coverslips were mounted with N-Propylgallate.

### Assessment of chromatin condensation and immunofluorescence

Hippocampal slices not treated with PI were fixed with PF 4% for 2 h and washed in PBS. After that, they were removed from the membrane inserts and placed in 24 well plates where they were permeabilized with 1% Triton X-100 in PBS for 2 h. Free floating slices were then incubated with 1% BSA in PBS for 2 h and incubated with primary antibodies in 1% BSA overnight at 37°C. Primary antibodies used include anti-rabbit CHOP/GADD153 (1:100; Santa Cruz) anti-mouse TUJ-1 (1:100; Sigma), anti-rabbit CHIP (1:100; Santa Cruz), anti-rabbit cleaved caspase-3 (1:100; Cell Signaling), anti-rabbit p53 (1:100, Santa Cruz). Following washes with PBS, tissues were incubated for 1 h at room temperature with Alexa Fluor 488-conjugated goat anti-rabbit, Alexa Fluor 555-conjugated goat anti-mouse antibodies (Invitrogen) diluted in PBS (1:200) plus TO-PRO3 (1:1000, Sigma) for nuclear staining. Tissues were then washed in PBS and mounted with N-propylgallate.

Slices were examined in a confocal microscope (Zeiss, LSM 510). Cells with condensed chromatin were counted in CA1 at 40x of magnification, in three distinct fields for each slice. Values represent mean percentages for slices under various treatments.

### Western blotting

Hippocampal slices were rinsed with PBS and then homogenized on ice in RIPA lysis buffer containing 1% TritonX-100, 1% DOC, 1% NP-40, NaCl 150 mM, TrisHCl 10 mM, EDTA 5 mM, SDS 0.1%, PMSF (10 mg/mL), pepstatin (1 mg/mL), aprotinin (2 mg/mL), leupeptin (2 mg/mL), NaF (22 mg/mL) and sodium ortovanadate (92 mg/mL). Lysates were centrifuged at 12,000 g for 15 min at 4°C. Protein concentration in the supernatant was determined with by Lowry protein assay. In a 10% SDS–polyacrylamide gel, 30 μg of protein was applied per lane for electrophoresis. After that, gel was transferred to nitrocellulose membranes (Bio-Rad) and processed for western blotting. First, membrane was blocked with 5% milk in T-TBS buffer (0.1% Tween in 20 mM Tris-HCl/137 mM NaCl; pH 7.3), then overnight with primary antibodies: anti-goat BIP/GRP78 (1:500, Santa Cruz); anti-rabbit phosphorylated eIF2-α (1:1000, Bioscience anti-rabbit eIF2-α (1:1000, Santa Cruz); anti-rabbit CHIP (1:1000, Santa Cruz) or anti-rabbit ERK-2 (1:2000, Santa Cruz). Washed membranes were incubated with an HRP-conjugated secondary anti-antibody for 1 h and revealed with the ECL Western Blotting Analysis reagent (Amersham Biosciences). Optical density on the blots was measured with ImageJ Software.

### Statistics

Values are expressed as the mean ± S.E.M. Statistical significance was assessed with One-Way ANOVA followed by Bonferroni's multiple comparison post-test. Each experiment represents a pool of hippocampal slices obtained from four rats of the same litter. Four to six slices were used for every condition of treatment and/or infection. We analyze three independent experiments obtained from three different litters for statistics.

## Results

### Tunicamycin induces UPR in hippocampal slices

**Figure 1:**
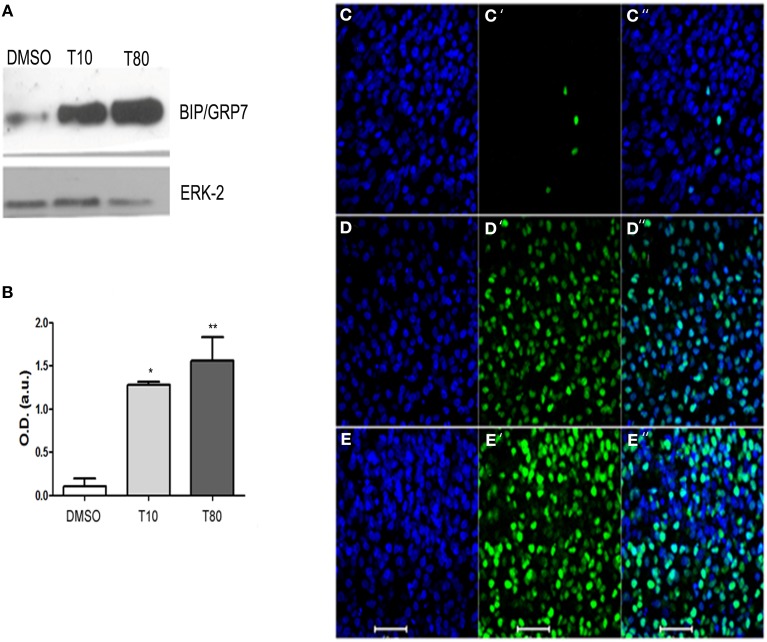
**Tunicamycin triggers unfolded protein response in hippocampal slices**. **(A)** Western Blot for BIP/GRP78 after treatment with DMSO (vehicle) or Tunicamycin 10 μg/mL (T10) and 80 μg/mL (T80) for 24 h. Western blot for ERK was used as loading control. **(B)** Quantification of optical density (O.D). Values represent ± S.E.M. compared to vehicles ^*^p < 0.1; ^**^p < 0.05. ANOVA was performed followed by Bonferroni's post-test. *P* < 0.01, C.I.: 99.9%, *N* = 4 independent experiments. **(C–E)** Immunofluorescence for CHOP/GADD153 (green) and counterstaining of DNA with TO-PRO3 (blue) after Tunicamycin treatment at 10 **(D)** or 80 μg/mL **(E)** or vehicles **(C)**. Note a nuclear localization of CHOP/GADD153. Scale bar: 20 μm.

To confirm that treatment with tunicamycin induces endoplasmic reticulum stress and activates the UPR in hippocampal cultures, protein extracts from slices maintained in the presence of tunicamycin at either 10 or 80 μg/mL were processed for western blot. We examined the content of the ER resident chaperone BiP/GRP78, the increased expression of which is a hallmark of UPR (Jäger et al., 2012). Figures 1A,B shows that both 10 or 80 mg/mL of tunicamycin efficiently increased the levels of BiP/GRP78. We also examined CHOP/GADD153, the pro-apoptotic component of the UPR. Tunicamycin also increased the levels of CHOP/GADD153 (Figures 1D,E) when compared to control (Figure 1C). Besides the increased immunolabeling, CHOP/GADD153 co-localized with the nuclear tracer TOPRO3, consistent with the function of CHOP/GADD153 as a transcription factor activated during UPR. These results confirmed that Tunicamycin induces endoplasmic reticulum stress and activates the UPR in hippocampal slices.

### Tunicamycin induces cell death in hippocampal slices

**Figure 2:**
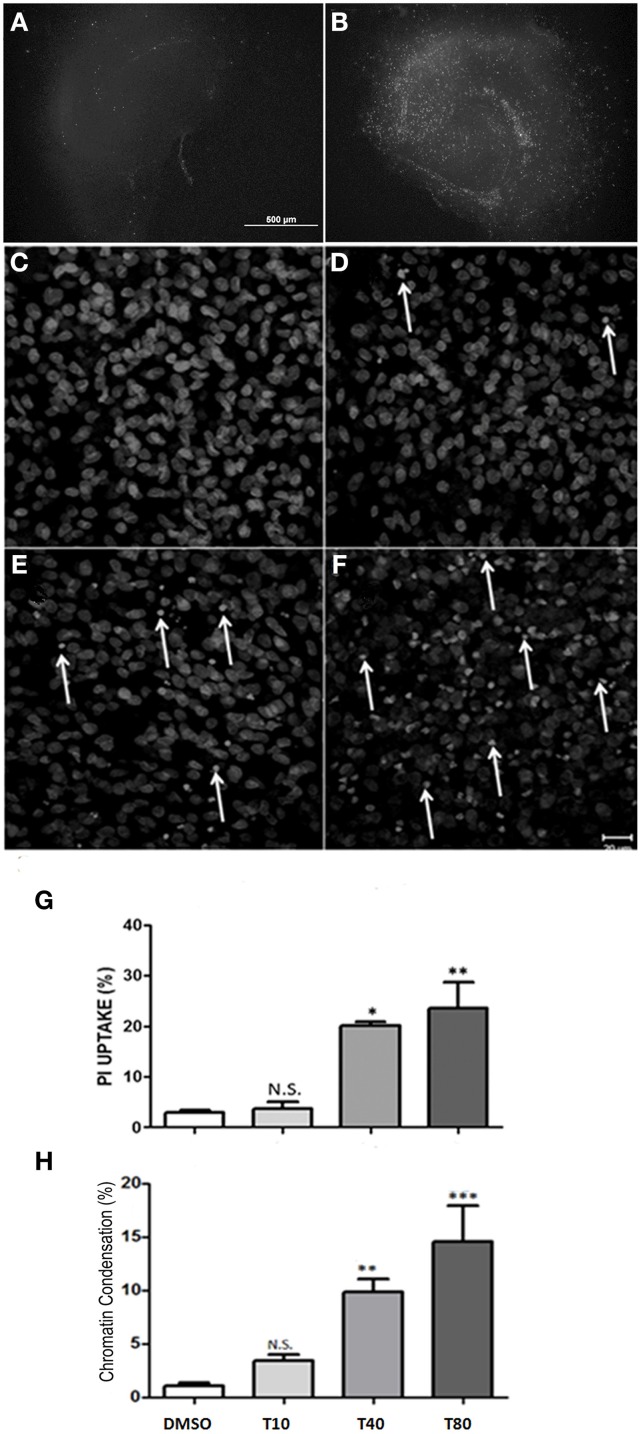
**Tunicamycin induces necrosis and apoptosis in hippocampal slices in a dose-dependent way**. Representative photomicrographies at 4x magnification shows propidium iodide (PI) uptake following 24 h of Tunicamycin treatment at 80 μg/mL. **(A)** DMSO, **(B)** Tunicamycin at 80 μg/mL; scale bar: 500 μm. **(C–F)** Photomicrographies of fixed slices stained with nuclear marker TO-PRO3 following Tunicamycin treatment at 10 **(D)**, 40 **(E)** and 80 μg/mL **(F)** for 24 h at 40x magnification compared to control **(C)**. Arrows point nuclei with apoptotic morphology. **(G)** Quantification of PI fluorescence percentage relative to maximum fluorescence intensity values of each different dose compared to control. Values represent ± S.E.M. compared to vehicle. One-Way ANOVA was performed followed by Bonferroni's post test. C.I.: 95%; *p* < 0.05; *N* = 3 independent experiments. **(H)** Quantification of chromatin condensation in treated slices compared to vehicles ^*^p < 0.1; ^**^p < 0.05; ^***^p < 0.0001. *P* < 0.01, C.I.: 99%, *N* = 4 independent experiments.

To analyze cell death in hippocampus after UPR activation, hippocampus slices were maintained *in vitro* for 13 days and then incubated with tunicamycin at 10, 40, 80 or 120 μg/mL. After 24 h in the presence of tunicamycin we quantified necrotic cell death by the uptake of PI in non-fixed hippocampal slices. As shown by Kosuge et al. (2008), Tunicamycin treatment at 40, 80 or 120 μg/mL were able to induce cell death in hippocampal slices after 24 h of treatment, especially at the DG and CA1 region (Figures 2A,B). Tunicamycin at 80 μg/mL promoted severe ER stress and was chosen for subsequent molecular approaches (Figure 2B). We also estimated cell death through the identification of nuclei with chromatin condensation, usually equated with apoptosis. For this analysis, CA1 was chosen as a region of interest, although similar phenotypic patterns were observed in other hippocampal subregions (data not shown). Figures 2C–F shows photomicrographs of CA1 of hippocampal slices maintained either with Tunicamycin Figures 2D–F or control medium (Figure 2C) for 24 h (from day 13 to 14). Cells with condensed chromatin were counted in CA1, revealing a dose-dependent effect of Tunicamycin. A similar increase in the proportion of cell death detected either by the quantification of PI uptake or chromatin condensation was observed following higher doses of Tunicamycin (compare Figures 2G,H).

### Overexpression of CHIP prevents ER stress-induced cell death in hippocampal slices

**Figure 3:**
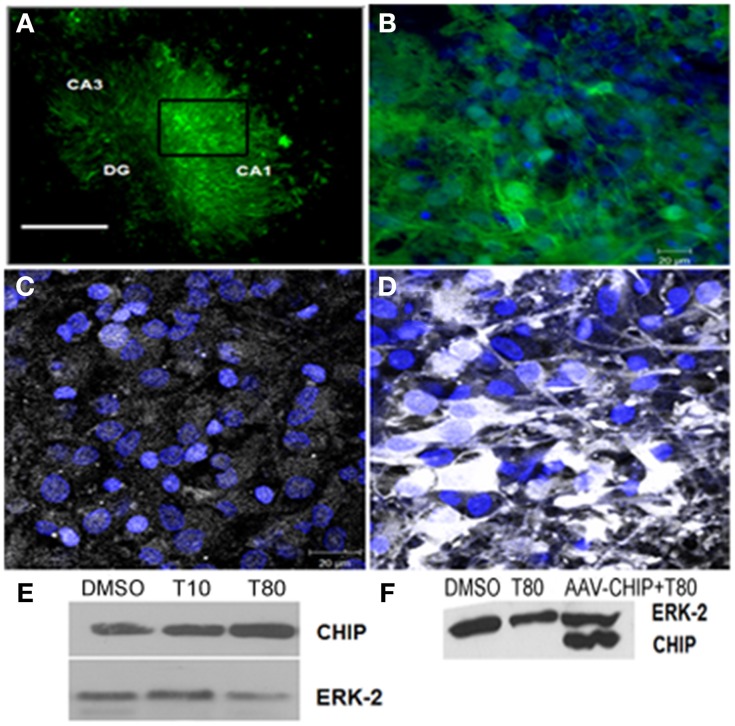
**Transgene distribution in hippocampal slices after rAAV8 infection**. Hippocampal slices were infected with rAAV8-GFP or rAAV8-CHIP and maintained for 14 days *in vitro*. **(A)** Representative photomicrography of slice infected in day *in vitro* 0 (DIV0) 14 days after rAAV8-GFP infection. 4x Magnification; scale bar: 500 μm. **(B)** Higher magnification (40x) of selected region in **(A)**. GFP fluorescence (green) and TOPRO3 (blue). **(C,D)** Immunofluorescence for CHIP (white) counterstained with TO-PRO3 (blue) comparing non-infected **(C)** with rAAV8-CHIP infected slice **(D)**; 63x magnification, scale bar: 20 μm. **(E)** Western blot for CHIP of Tunicamycin treated slices at 10 (T10) or 80 μg/mL (T80) compared to vehicles (DMSO). **(F)** Western blot comparing samples infected with rAAV8-CHIP to non-infected slices. **(E,F)** Western blot for ERK-2 was used as loading control.

To test whether overexpression of CHIP alters cell death induced by ER stress in hippocampus slices we used adeno-associated virus serotype 8 (rAAV8) as a carrier. First we assess the transduction efficiency 14 days after infection, we used GFP as a control transgene (rAAV8-GFP) and examined the distribution of its fluorescence. Figures 3A,B shows GFP fluorescence in all hippocampus regions indicating a very efficient transduction of the tissue in our experimental condition. rAAV8-CHIP induced an increase in the content of CHIP after 14 days of infection (Figures 3C,D), which was confirmed by Western blots (Figure 3F). There was also a trend of increased CHIP content after treatment with Tunicamycin at 80 μg/mL (Figure 3E).

**Figure 4:**
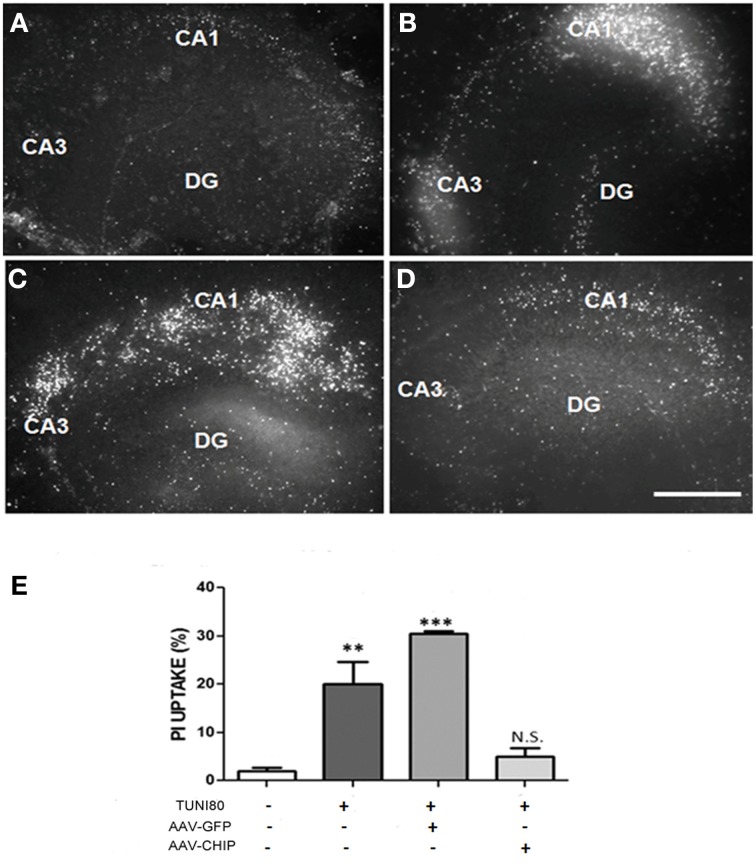
**Overexpression of CHIP attenuates PI uptake in all hippocampal subregions induced by Tunicamycin**. Hippocampal slices were infected with rAAV8-GFP or rAAV8-CHIP. After 13 days *in vitro*, the slices were incubated with Tunicamycin at 80 μg/mL for 24 h. Freshly slices were incubated with PI. PI uptake of vehicle treated slice **(A)**, Tunicamycin at 80 μg/mL **(B)**, rAAV8-GFP + Tunicamycin at 80 μg/mL **(C)**, rAAV8-CHIP + tunicamycin at 80 μg/mL **(D)**. 4x Magnification, scale bar: 500 μm. **(E)** Quantification of PI fluorescence percentage relative to maximum fluorescence values ^**^p < 0.05; ^***^p < 0.0001. Values represent ± S.E.M. comparing all groups to vehicles. Confidence Interval: 99%; *p* < 0.01; *N* = 4 independent experiments.

After 13 days post infection with rAAV8-CHIP or rAAV8-GFP, hippocampal slices were treated with Tunicamycin 80 μg/mL for 24 h. PI uptake was similar in both rAAV8-GFP infected (Figure 4C) and uninfected hipppocampal slices (Figure 4B). In contrast, rAAV8-CHIP reduced PI uptake to levels comparable with untreated slices (Figures 4A,D). These data indicate that overexpression of CHIP blocked necrosis induced by ER stress (Figure 4E).

Importantly, untreated tissue infected with rAAV8-CHIP or rAAV8-GFP did not show changes in the uptake of PI when compared to control slices, indicating that infection with the viral vector by itself does not significantly modulate cell death in this model (Figure S1).

**Figure 5:**
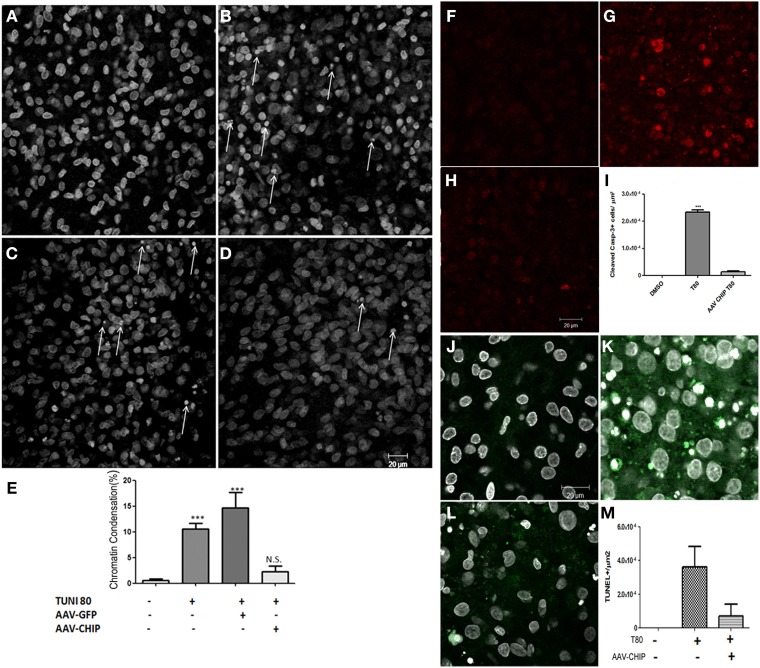
**Overexpression of CHIP reduces apoptosis in hippocampal slices treated with Tunicamycin**. Hippocampal slices were infected with rAAV8-GFP or rAAV8-CHIP. After 13 days *in vitro*, the slices were incubated with Tunicamycin at 80 μg/mL for 24 h. Analysis of chromatin condensation with TO-PRO3 staining **(A–D)** of slices treated with vehicle **(A)**, Tunicamycin at 80 μg/mL **(B)**; rAAV8-GFP + Tunicamycin at 80 μg/mL **(C)**; rAAV8-CHIP + Tunicamycin at 80 μg/mL **(D)**. 40x Magnification, Scale bar: 20 μm. **(E)** Quantification of chromatin condensation in treated/infected slices compared to vehicles. *P* < 0.01, C.I.: 99.9%, *N* = 3 independent experiments. **(F–H)** Immunofluorescence for cleaved caspase 3 (in red) after Tunicamycin treatment **(G)**, vehicle **(F)** and rAAV8-CHIP + Tunicamycin treated **(H)**. 63x Magnification, Scale bar: 20 μm. **(I)** Quantification of positive cells for cleaved caspase 3. *p* < 0.01, C.I.: 99.9%, *N* = 2 independent experiments. **(L)** TUNEL labeling (green) counterstained with TO-PRO3 (white). **(J)** Vehicle, **(K)** Tunicamycin and **(L)** rAAV8-CHIP + Tunicamycin. 63x Magnification, Scale bar: 20 μm. **(M)** Quantification of TUNEL positive cells. *N* = 2 independent experiments ^***^p < 0.0001.

Apoptotic cell death was also estimated after overexpression of CHIP. Slices treated with tunicamycin at 80 μg/mL had an increased percentage of cells with condensed chromatin in CA1 compared to control slices, while overexpression of CHIP reduced the proportion of apoptotic nuclei (Figure 5). rAAV8-GFP had no effect. rAAV8 infection by itself also did not induce chromatin condensation (Figure S2). We further examined immunofluorescence for cleaved caspase-3 and TUNEL as markers of apoptosis. Tunicamycin increased the number of positive cells detected by either method, whereas rAAV8-CHIP abrogated this effect (Figures 5F–M). The data altogether indicate that overexpression of CHIP in hippocampal tissue attenuates both necrosis and apoptosis induced by ER stress.

### Overexpression of CHIP prevents activation of pro-apoptotic mediators following UPR activation

**Figure 6:**
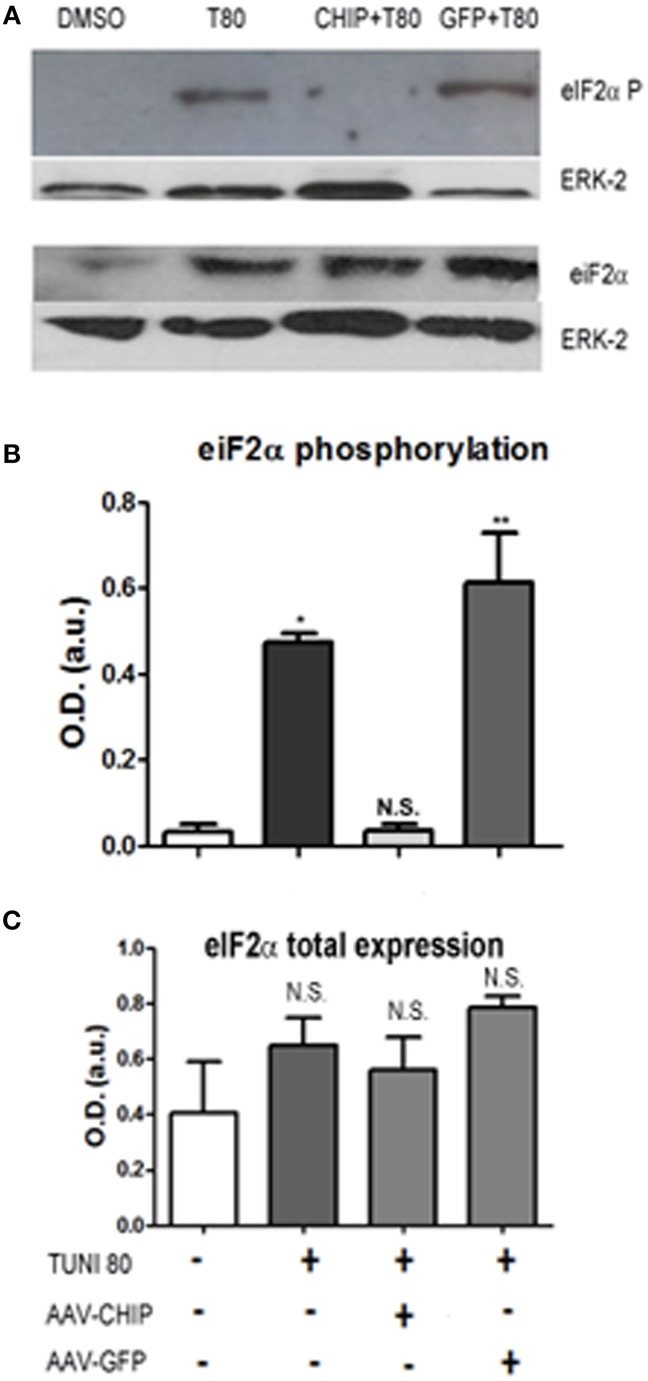
**Overexpression of CHIP reduces eIF2-α phosphorylation after Tunicamycin treatment**. Western Blot **(A)** for phosphorylated eIF2-α (upper) and total eIF2-α expression (down) comparing vehicles (DMSO) to treated/infected slices. ERK-2 was used as a loading control. **(B,C)** Quantification for optical density (O.D) ^*^p < 0.1, ^**^p < 0.05. Values represent ± S.E.M. compared to vehicles. *P* < 0.1, C.I.: 90%, *N* = 3 independent experiments.

The elongation factor eIF2-α is a substrate of PERK, one of the ER stress sensors. We found that at 24 h of treatment, Tunicamycin induced a significant increase in eIF2-α phosphorylation, consistent with activation of the UPR. In turn, overexpression of CHIP, but not of GFP, abrogated eIF2-α phosphorylation as shown in Figure 6.

**Figure 7:**
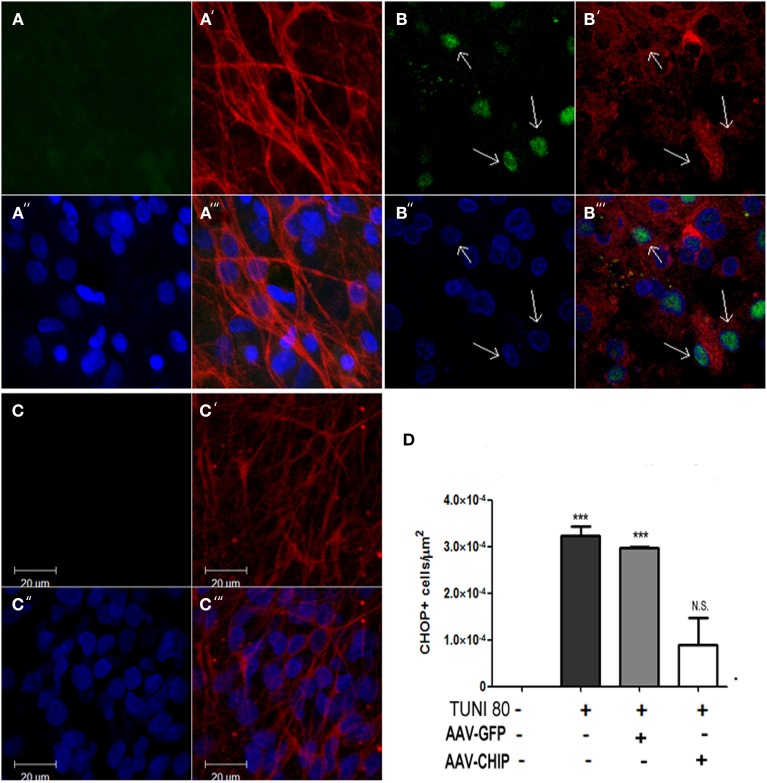
**Overexpression of CHIP diminishes CHOP/GADD153 positive neurons after Tunicamycin treatment**. Immunofluorescence for CHOP (green) and TUJ-1 (red) counterstained with nuclear marker TO-PRO3 (in blue). **(A–A″′)** Vehicle treated slice. **(B-B″′)** Tunicamycin treated slice. **(C-C″′)** rAAV8-CHIP + Tunicamycin treated slice. 63x Magnification, scale bar: 20 μm. Arrows point CHOP+ neurons in CA1 region. **(D)**. Quantitative analysis of CHOP/GADD153 + cells/area ^***^p < 0.0001. Values represent ± S.E.M. compared to vehicles. *P* < 0.01, C.I.: 99.9%, *N* = 3 independent experiments.

CHOP/GADD153 is a classical hallmark of apoptotic signalization triggered after UPR. To test whether CHIP overexpression alters the upregulation of CHOP/GADD153 induced by Tunicamycin, we immunostained slices infected with either rAAV8-GFP or CHIP, and estimated the amount of CHOP/GADD153 positive cells. In Figure 7, we show that overexpression of CHIP, but not GFP, prevented Tunicamycin-induced upregulation of CHOP/GADD153. Immunolabeling for TUJ-1 in CA1 region suggests neurodegeneration following Tunicamycin, as shown by the morphology of pyramidal cells stained with TUJ-1. Overexpression of CHIP, on the other hand, prevented such alterations. Taken together, these results suggest that the neuroprotection by CHIP is accompanied by a reduction in the activity of the pro-apoptotic UPR pathway.

**Figure 8:**
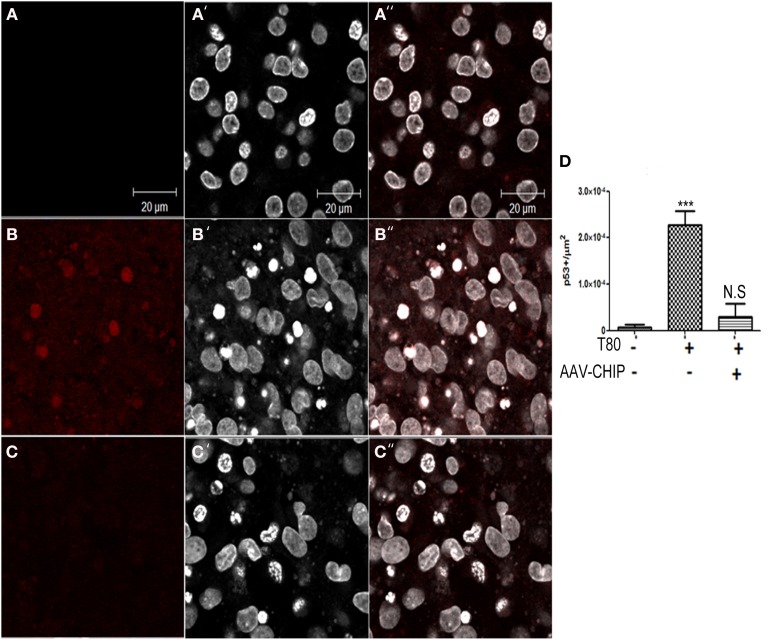
**Overexpression of CHIP diminishes p53 positive cells in hippocampal slices treated with Tunicamycin**. **(A–C)** Immunofluorescence for p53 (in red) counterstained with nuclear marker TO-PRO3 (in white) shows increased expression in treated slices **(B–B″)** compared to vehicles **(A–A″)** and rAAV8-CHIP+ Tunicamycin treated slices **(C–C″)**. **(D)** Quantitative analysis of p53+ cells/area ^***^p < 0.0001. Values represent ± S.E.M. compared to vehicles. *P* < 0.01, C.I.: 99%, *N* = 3 independent experiments.

Previous work has shown that p53 is also a downstream player associated with UPR activation (Li et al., 2006). We examined the content of p53 by immunofluorescence, and found that Tunicamycin increased the number of p53 positive cells in hippocampal tissue (Figure 8), whereas rAAV8-CHIP prevented the upregulation of p53.

### Upregulation of BIP/GRP78 induced by Tunicamycin was not altered by overexpression of CHIP

**Figure 9:**
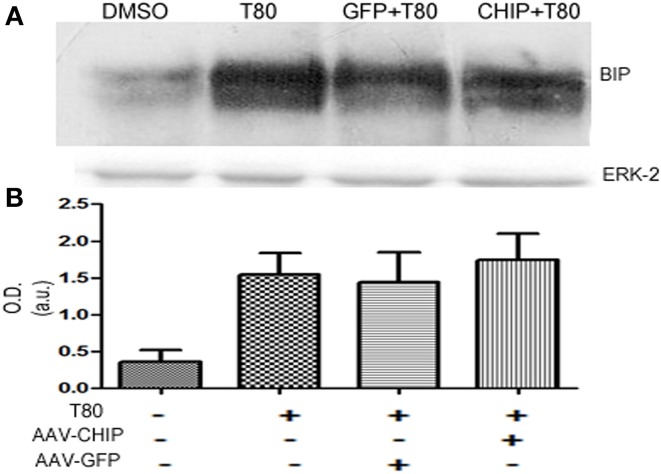
**BIP/GRP78 expression is not altered after Tunicamycin treatment in slices overexpressing CHIP**. **(A)** Western blots for BIP/GRP78; ERK-2 used as loading control. **(B)** Quantification for optical density (O.D). Values represent ± S.E.M. compared to vehicles. *P* < 0.05, C.I.: 95%, *N* = 3 independent experiments.

Western blot for the chaperone BIP/GRP78 showed that Tunicamycin increased the content of BIP/GRP78 (Figure 9), an effect that was not changed by either rAAV8-GFP or rAAV8-CHIP (Figure 9). These results suggest that overexpression of CHIP did not prevent ER stress, nor the activation of the adaptative UPR in hippocampal slices following Tunicamycin treatment.

## Discussion

In this work we found that overexpression of CHIP prevented cell death induced by ER stress in the hippocampus. In addition, CHIP attenuated the phosphorylation of eIF2-α as well as the upregulation of both CHOP and p53, which are part of the ER stress death response. However, overexpression of CHIP did not prevent the increase of BiP/GRP78, which is an adaptative response induced by ER stress. These data indicate a neuroprotective role for CHIP upon UPR signaling.

Previous work showed that tunicamycin induces PI uptake in hippocampal slices (Kosuge et al., 2008), while the present study adds the evidence that markers of apoptotic cell death were also increased. However, a low concentration of Tunicamycin induced an increase of both BiP and CHOP expression without cell death, which is consistent with the predominance of an adaptive response to moderate ER stress.

Consistent with our results, overexpression of CHIP enhanced cell viability after treatment with Tunicamycin in HeLa cells (Dikshit and Jana, 2007). However, the mechanisms of cytoprotection by CHIP were not clarified. Cell death induced by ER stress may be independently triggered by at least two pathways: one mediated by p53 and other mediated by CHOP (Li et al., 2006). In the present study, overexpression of CHIP prevented the upregulation of both p53 and CHOP after treatment with tunicamycin, which suggests that neuroprotection conferred by CHIP may be due to attenuation of both pathways. Recent work has presented data consistent with degradation of p53 by CHIP E3 ligase activity, thus preventing cell death (Naito et al., 2010), which may explain the neuroprotective effect. However, although CHOP is degraded by the ubiquitin proteasome system (Ohoka et al., 2007), there are no reports of effects of CHIP upon CHOP degradation.

CHIP also attenuated phosphorylation of eIF2-α induced by tunicamycin. The role of eIF2-α phosphorylation upon neurodegeneration induced by ER stress is controversial (Moreno and Tiffany-Castiglioni, 2014). An inhibitor of dephosphorylation of eIF2-α prevented cell death induced by ER stress, indicating that P-eIF2-α is cytoprotective (Boyce et al., 2005). In contrast, phosphorylated eIF2-α was also identified as essential for neurodegeneration (Moreno et al., 2012). It has been described that PERK can induce transcription of CHOP mediated by phosphorylation of eIF2-α (Oyadomari and Mori, 2004). Thus, the attenuation of eIF2-α phosphorylation, promoted by CHIP, may be linked to downregulation of CHOP.

**Figure 10:**
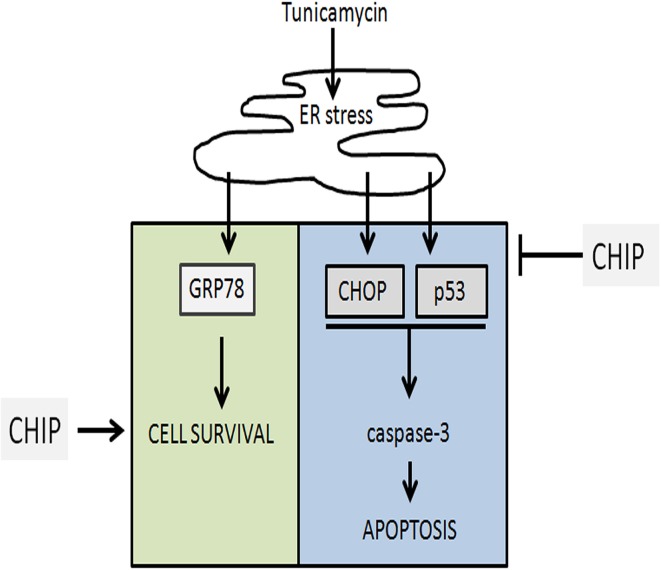
**Representation of the final conclusion**. Tunicamycin treatment induces ER stress which activates UPR and upregulates GRP78, CHOP, p53. Overexpression of CHIP prevented cell death blocking upregulation of CHOP, p53, and caspase-3 activation, while maintain upregulation of GRP78 (BiP) and cell survival.

Overexpression of CHIP also blocked the activation of caspase-3 induced by Tunicamycin in hippocampus. The intrinsic pathway of activation of caspases is modulated by the Bcl-2 family (McCullough et al., 2001). CHOP blocks transcription of the antiapoptotic gene Bcl-2, while p53 activates transcription of proapoptotic BH3 proteins such as PUMA and NOXA, as well as Bax (Oyadomari and Mori, 2004; Li et al., 2006; Moldoveanu et al., 2014). In this work CHIP prevented the increase of p53 and CHOP, suggesting that the citoprotective action of CHIP is upstream of the expression of Bcl-2 family and activation of caspases (Figure 10).

The ER chaperone BiP/GRP78 promotes protein folding and directs misfolded proteins to degradation. These functions are important to maintain ER homeostasis. Increased expression of BiP/GRP78 is critical for protective adaptation to ER stress. Overexpression of CHIP did not affect the increase of BiP induced by Tunicamycin, showing that CHIP does not prevent activation of the UPR. This, together with the reduction of P-eIF2-α, CHOP, and p53, indicated that CHIP targeted the cell death components of the UPR but not its adaptive response (Figure 10).

CHIP binds to client proteins of HSP70 and HSP90, and ubiquitinates proteins, both in cytosol and nucleus, but it has not been found in the ER. Our results suggest that overexpression of CHIP affects protein quality control in the ER as well, thus attenuating ER stress, which is consistent with cell free system data that suggests that CHIP modulates ERAD (Matsumura et al., 2013). This corroborates the view that mechanisms of proteostasis in distinct cellular compartments may be interconnected. Given that ER stress and protein misfolding are associated with various neurodegenerative diseases, CHIP may emerge as a candidate for clinical intervention in such conditions.

## Author contributions

Felipe Cabral Miranda and Juliana Adão-Novaes have designed and performed all experiments, analyzed, and interpreted data. Felipe Cabral Miranda, William W. Hauswirth, Rafael Linden, Petrs-Silva, and Luciana B. Chiarini. have contributed to the design of experiments and provided funding for acquisition of data. All authors have participated in the writing process of the manuscript and critically analyzed data improving the quality of the work for publication.

### Conflict of interest statement

The authors declare that the research was conducted in the absence of any commercial or financial relationships that could be construed as a potential conflict of interest.

## Supplementary material

Figure S1 **rAAV8-GFP (B) or rAAV8-CHIP (C) infected slices does not exhibit significant alterations in PI uptake compared to control (A)**. PI uptake comparison showed no statistical difference. N.S.: no significance C.I.: 90%, *N* = 3 independent experiments.

Figure S2 **rAAV8-GFP (B) or rAAV8-CHIP (C) infected slices does not present alterations in chromatin condensation compared to control (A)**. N.S.: no significance C.I.: 90%, *N* = 3 independent experiments.
