# Supplementary figures and images for: CHIP, a carboxy terminus HSP-70 interacting protein, prevents cell death induced by endoplasmic reticulum stress in the central nervous system
Source: Front Cell Neurosci. 2015 Jan 9;8:438. doi: 10.3389/fncel.2014.00438 (PMC4288139; doi:10.3389/fncel.2014.00438)

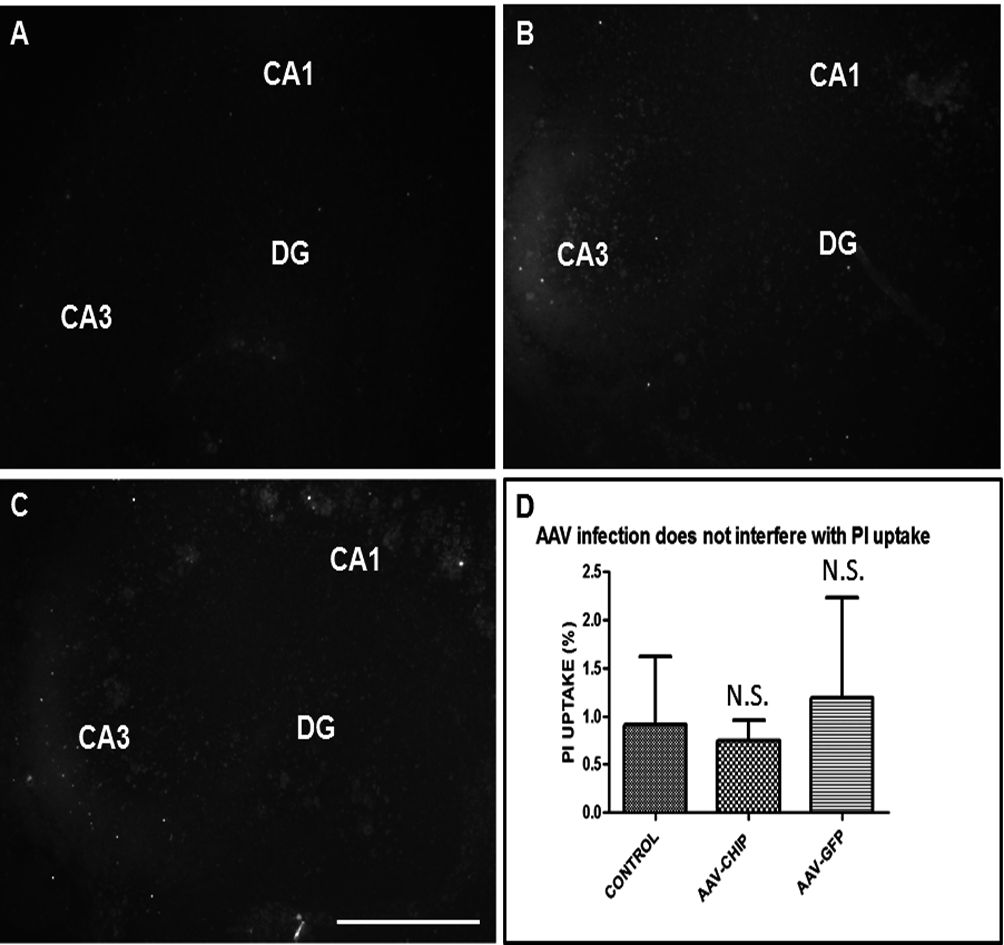

Supplement: Figure S1 — rAAV8-GFP (B) or rAAV8-CHIP (C) infected slices does not exhibit significant alterations in PI uptake compared to control (A). PI uptake comparison showed no statistical difference. N.S.: no significance C.I.: 90%, N = 3 independent experiments. [file Image1.TIF]

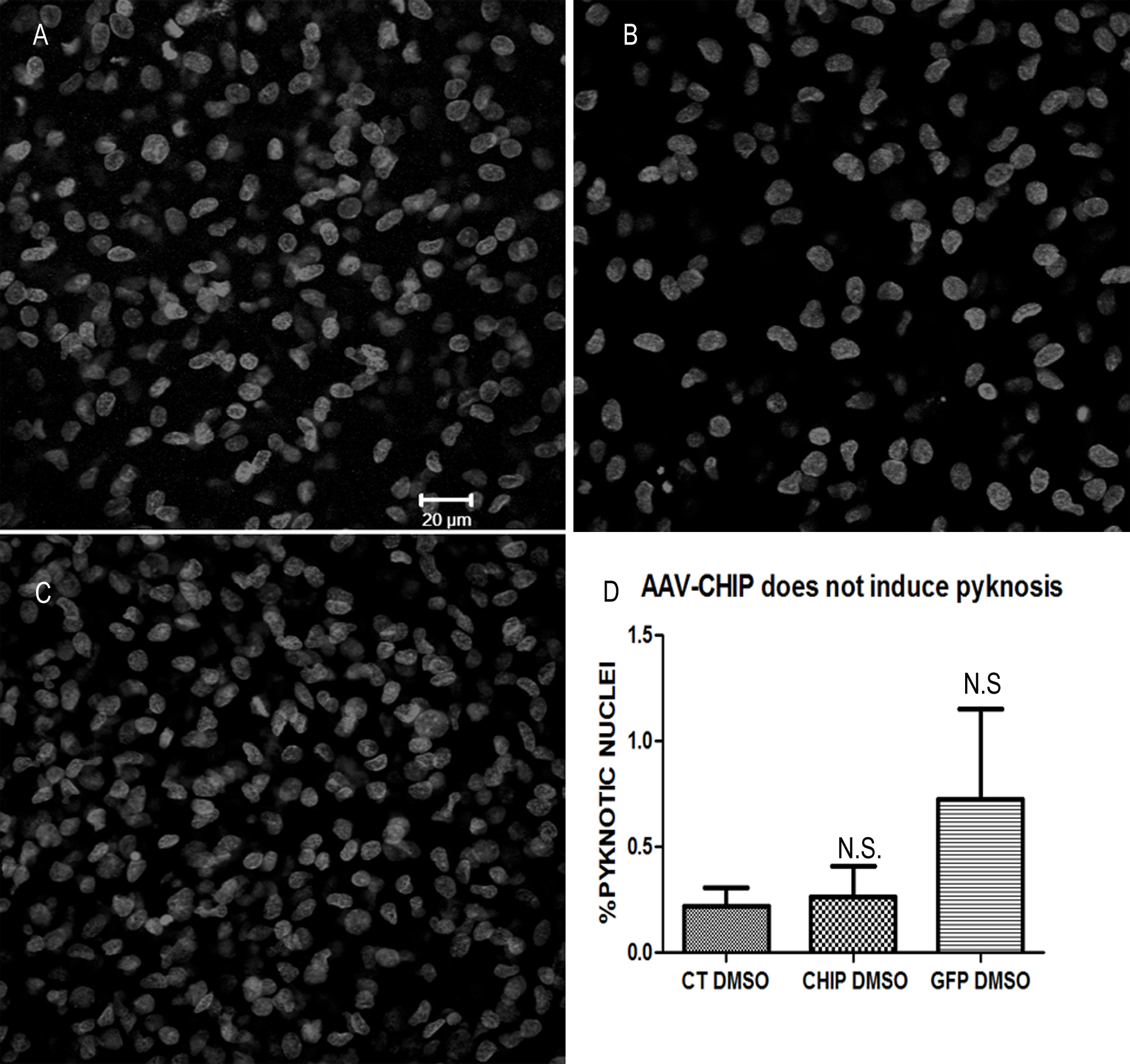

Supplement: Figure S2 — rAAV8-GFP (B) or rAAV8-CHIP (C) infected slices does not present alterations in chromatin condensation compared to control (A). N.S.: no significance C.I.: 90%, N = 3 independent experiments. [file Image2.TIF]
